# The Effects of Autonomy-Enhancing Therapy for Eating Disorders: Protocol for a Single-Case Experimental Multiple Baseline Design

**DOI:** 10.2196/87349

**Published:** 2026-09-22

**Authors:** Nicky Bosman, Joyce Maas, Marcel van Assen, Mladena Simeunovic-Ostojic, Marrie Bekker

**Affiliations:** 1 Center for Eating Disorders, GGZ Oost Brabant, Wesselmanlaan 25A, Helmond, 5707 HA, The Netherlands, 31 621177294; 2 Department of Clinical, Neuro- and Developmental Psychology, Faculty of Behavioral and Movement Sciences, VU University, Amsterdam, The Netherlands; 3 Department of Medical and Clinical Psychology, Center of Research on Psychological disorders and Somatic diseases, Tilburg University, Tilburg, The Netherlands; 4 Department of Methodology and Statistics, Tilburg University, Tilburg, The Netherlands

**Keywords:** autonomy-connectedness, eating disorders, autonomy-enhancing therapy, anorexia nervosa, bulimia nervosa

## Abstract

**Background:**

Eating disorders (EDs) remain challenging to treat effectively, with low recovery percentages and high relapse rates. This study protocol explores autonomy-enhancing therapy as a therapeutic adjunct to existing day care treatment in the last phase of treatment. Autonomy-enhancing therapy is aimed at improving autonomy-connectedness: the capacity for self-steering while connected to others, which encompasses self-awareness, sensitivity to others, and the capacity for managing new situations. Higher autonomy-connectedness has been associated with improved ED recovery and lower relapse rates, yet little is known about the effectiveness of autonomy-enhancing therapy in ED populations.

**Objective:**

This study examines the effectiveness of autonomy-enhancing therapy in the last phase of a multidisciplinary day care program for anorexia and bulimia nervosa.

**Methods:**

A combined single-case experimental design and repeated measures design will be used, involving participants who are in the last phase of day care treatment for anorexia or bulimia nervosa (n=22-30). Participants will receive weekly autonomy-enhancing therapy sessions focused on personal autonomy-related goals and themed group discussions. The primary outcomes (ED symptoms and autonomy-connectedness) will be assessed weekly using the Eating Disorder Examination Questionnaire–Short (EDE-QS) and a short-term version of the Autonomy-Connectedness Scale (ACS-30-ST). Secondary outcomes (depression, anxiety, self-esteem, and quality of life) will be assessed every 3 months using the Symptom Checklist (SCL-90), Rosenberg Self-Esteem Scale (RSES), and World Health Organization Quality of Life–Brief Version (WHOQOL-BREF). Throughout the treatment, we expect improvements in autonomy-connectedness and a decrease in ED symptoms, with autonomy-connectedness mediating treatment success. Additionally, increased self-esteem and quality of life and reduced depression and anxiety are expected after treatment. Visual analysis of the single-case experimental design data and mixed models will be used to evaluate treatment effectiveness, with mediation analyses exploring the role of autonomy-connectedness.

**Results:**

Data collection started in March 2024. As of September 2026, 18 participants have completed the study, and 6 are enrolled. An additional 7 participants have been recruited. Data collection is expected to be finalized in February 2027; results are expected to be published in the second half of 2027.

**Conclusions:**

This study addresses the need for improved ED treatments by investigating autonomy-enhancing therapy as a potentially valuable adjunct to existing care. Targeting autonomy-connectedness may contribute to reductions in ED symptoms, as well as to broader psychological recovery. Strengths of this study include its practice-based design and the use of a single-case experimental design, allowing a detailed examination of treatment processes. Limitations include the absence of a control condition and the limited follow-up period, which may not fully capture relapse during the period of highest relapse risk. If promising, this study may provide a foundation for future larger-scale controlled studies on autonomy-enhancing therapy in ED treatment.

## Introduction

### Background

Eating disorders (ED) in adults are notoriously difficult to treat. Although guidelines recommend several well-established treatments [1], it remains unclear which of these is most effective, and no treatment for anorexia nervosa (AN) currently meets the highest level of evidence [2] (ie, level I evidence according to the Oxford Centre for Evidence-Based Medicine criteria [3]). Currently, only about 50% of patients with bulimia nervosa (BN) and 40% of individuals with AN fully recover after 5 years [4], with relapse rates between 22% and 51% [5]. The highest relapse risk occurs 4 to 6 months after treatment [4]. The debilitating nature of ED makes these high relapse and low recovery rates particularly concerning [6].

At the same time, there is increasing recognition that EDs involve more than eating and weight-related symptoms alone. Evidence suggests that disturbances in self-functioning and interpersonal functioning play an important role in the development and maintenance of these disorders [7]. Historically, EDs have been closely linked to disturbances in identity and the self [8]. Difficulties such as low awareness of needs [9] and poor self-concept clarity [10], paired with high self-criticism and self-objectification, may increase vulnerability to eating pathology by fostering reliance on external validation in defining identity and worth [9,11] and the need to control food and the body as a way to experience autonomy, competence, and identity [8].

Thus, improving treatment to aid transdiagnostic recovery, societal rehabilitation, and relapse prevention is crucial. Given the associations between autonomy-connectedness and ED symptoms and comorbidity, autonomy-connectedness could be a useful addition to therapy [12-14]. Autonomy-connectedness refers to the capacity to identify, express, and pursue one’s own needs, wishes, and values while maintaining meaningful and reciprocal relationships with others. It reflects both self-direction and interpersonal connectedness, capturing the degree to which individuals can identify and strive for their own needs, considering those of others. Autonomy-connectedness consists of 3 components: sensitivity to others’ wishes, needs, and opinions; self-awareness, the awareness of one’s own wishes, opinions, and needs and the capacity to express these in social interactions; and the capacity for managing new situations, understood as the inclination to explore, and feeling at ease in new situations [15]. Autonomy-connectedness is informed by attachment theory and neoanalytical object-relation theory on the development of gender identity [16], and it understands that healthy psychological functioning implies a strong self as well as strong connectedness to others [15]. Autonomy-connectedness deficits are an important underlying vulnerability factor for developing mental health problems [17].

Several considerations support the potential value of autonomy-connectedness: (1) autonomy-connectedness predicts ED recovery [12]; in a longitudinal study, recovered patients showed decreases in sensitivity to others and increases in the capacity for managing new situations compared with patients who did not recover; (2) autonomy-connectedness is transdiagnostic, which is relevant considering the high comorbidity in ED [18]; (3) autonomy-connectedness partly explains sex differences in ED symptom severity [13] and develops during the same life stages (puberty and young adulthood) in which EDs typically emerge [19], with the shift toward independence and separation contributing to sex-specific prevalence differences in mental health issues; (4) autonomy-connectedness promotes a healthy identity by addressing the ego-syntonic nature of EDs, which can contribute to high relapse rates due to strong identification with the disorder [20]; and (5) a well-founded autonomy-connectedness therapy protocol, autonomy-enhancing therapy, exists [21]. Each of these will be elaborated on in the following sections.

The first reason to include autonomy-connectedness in adult ED treatment is that it has been identified as one of the strongest predictors of recovery from ED after 1 year of treatment [12]. Kuijpers et al [12] found that patients who recovered initially had higher sensitivity to others and showed a significant decrease in sensitivity to others along with an increased capacity for managing new situations after treatment when compared with patients who did not recover. These findings indicate that recovered patients benefited from their ED treatment by gaining autonomy.

Second, autonomy-connectedness is a transdiagnostic construct associated with all major comorbidities typical of ED [18]. For example, autonomy-connectedness is linked to anxiety [14,22,23] and depression [24-29], which are characterized by high sensitivity to others paired with low self-awareness. Additionally, excessive sensitivity to others is linked to internalizing personality disorders, while low sensitivity to others and a high capacity for managing new situations are associated with antisocial personality disorder characteristics [30]. Comorbid diagnoses, particularly mood and anxiety disorders [31], may reinforce ED symptoms and can affect treatment efficacy [32]. Importantly, autonomy-enhancing therapy has already been shown to be similarly effective as group cognitive behavioral therapy in treating anxiety disorders and related comorbid symptoms [23]. Incorporating a transdiagnostic construct such as autonomy-connectedness in ED treatment may therefore not only support ED treatment but also enhance the likelihood of sustainable recovery through the concurrent treatment of comorbid conditions.

Third, autonomy-connectedness is an important factor in explaining observed sex differences in ED symptom severity. In a large, representative community sample, autonomy-connectedness differences between the sexes partially explained sex differences in ED symptom severity [13]. Men and women consistently differ in sensitivity to others, with women reporting much higher sensitivity to others than men [33]. These differences may stem from gendered attachment experiences, which in turn shape distinct adult identities and psychological vulnerabilities. Importantly, the development of autonomy-connectedness coincides with the life stage in which EDs typically emerge: puberty and young adulthood [19]. Adolescence marks the move toward changes in relationships with parents (growing independence), while adolescents’ relationships with peers become more and more important, which indicates that the “self” is still being developed. This is when the prevalence of anxiety, depression, and ED starts spiking in girls compared with boys [34], and the abovementioned differences in autonomy levels also emerge [33]. Interestingly, observed sex differences in ED symptom severity are substantially reduced when controlling for sex differences in autonomy-connectedness [13]. This suggests that autonomy-connectedness may partly account for sex differences in ED symptoms. These sex differences in autonomy-connectedness may arise from differing gendered attachment experiences for both sexes [8]. This finding suggests that it might not be solely “femaleness” that increases the likelihood of developing ED, but rather feminine attachment experiences.

Fourth, autonomy-connectedness relates to self-awareness and identity. ED, particularly AN, are often described as ego-syntonic phenomena (ie, the ED is seen as part of one’s true self, not an external illness that requires treatment) [20]. This ego-syntonic nature is suggested to contribute to high relapse rates [20]. Even if the current treatment effectively removes most ED symptoms, patients often struggle with identity and low empowerment afterwards, as the ED was intertwined with their sense of self [35]. Therefore, additional efforts are needed to rebuild a healthy self.

Finally, although a well-founded autonomy-connectedness therapy protocol, autonomy-enhancing therapy, exists [21], little is known about its effectiveness in ED treatment. Addressing this gap constitutes the need for the current study. For more details on autonomy-enhancing therapy, refer to the Methods section.

In summary, autonomy-connectedness appears to provide new insights into ED, and it is a promising and likely useful addition to ED treatment. Therefore, the study described in this protocol is aimed at testing the effectiveness and mechanisms of autonomy-enhancing therapy in the last phase of ED treatment for AN and BN.

### Hypotheses

Throughout autonomy-enhancing therapy, we expect (1) ED symptoms to decrease, (2) autonomy-connectedness to improve (ie, self-awareness and the capacity for managing new situations to increase, and sensitivity to others to decrease), and (3) autonomy-connectedness to mediate treatment success.

We will explore the differences between ED diagnoses (AN and BN) in relation to hypotheses 1 to 3.

Additionally, with respect to the secondary outcomes, we expect increased self-esteem and quality of life and reduced depression and anxiety at the posttreatment assessment compared with the pretreatment assessment.

## Methods

### Ethical Considerations

Ethics approval for the study protocol was obtained from the Medical Ethics Review Committee of VU Amsterdam (NL83503.018.23) and the internal research ethics committee of GGZ Oost Brabant. All participants received written and verbal information about the study and provided informed consent to participate. All participant data are being deidentified. Data collection takes place through ResearchManager EDC (ResearchManager), a secure program through which participants are assigned a unique participant number. Only the principal researcher has access to the key linking participant numbers to names and email addresses.

### Design

Two designs are combined: a single-case experimental design (SCED) and a repeated measures (RM) design. Given the vulnerable and highly specific population of patients with severe EDs, a SCED was considered more feasible and ethically appropriate than, for example, a randomized controlled trial, as it reduces the number of participants required and avoids withholding treatment from a control group.

A SCED multiple baseline design differentiates between time and intervention effects, with participants serving as their own controls. Experimental control is demonstrated when changes in outcome variables occur consistently after the introduction of the intervention across staggered baselines. Replication of this pattern across participants strengthens causal inference regarding intervention effects. The number of baseline measurements is randomized (ie, the number of measurements before the start of the intervention), varying from 5 to 8 weeks (ie, 5‐8 measuring points). Weekly measurements are taken for the duration of the assigned baseline plus the length of the intervention. The Eating Disorder Examination Questionnaire–Short (EDE-QS) [36] and the ACS-30-ST measure short-term ED symptoms and autonomy-connectedness characteristics, respectively.

**Figure 1.**
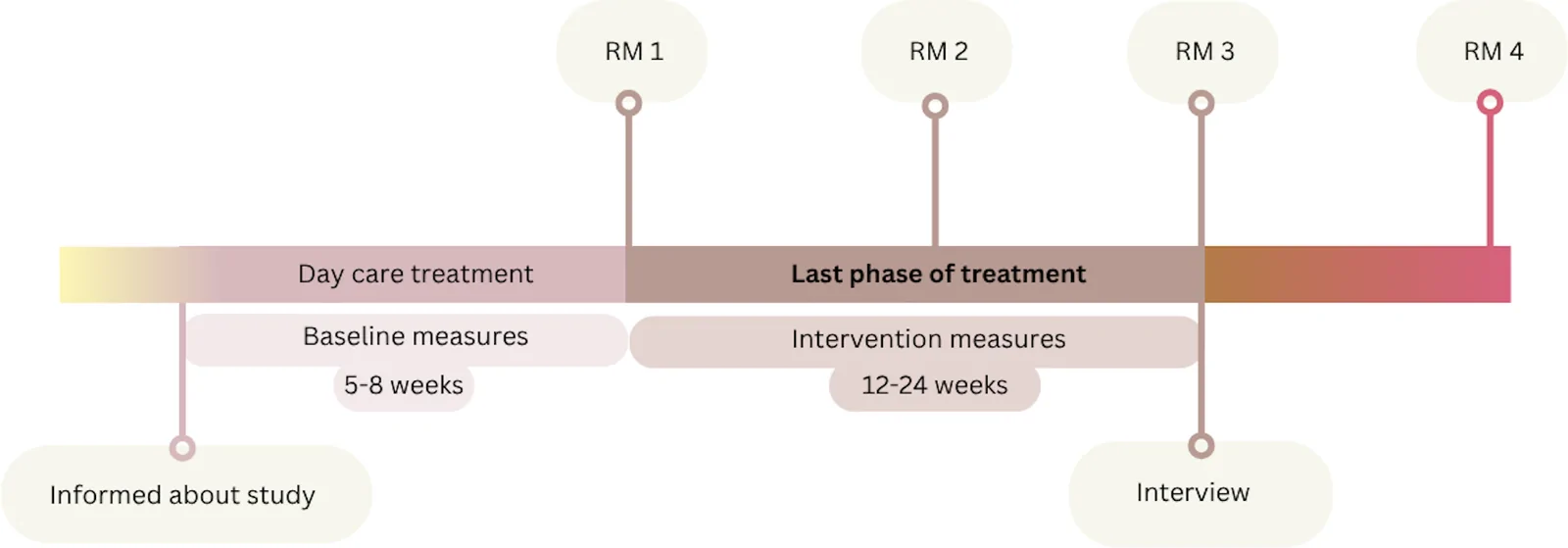
Study procedure flowchart. RM: repeated measures.

The RM design assesses long-term ED symptoms and autonomy-connectedness characteristics, as well as depressive and anxiety symptoms, self-esteem, and quality of life, every 3 months at the beginning, middle, and end of the intervention, along with a 3-month follow-up (4 measurements). After treatment, participants will also be interviewed about their experiences with autonomy-enhancing therapy. The interviews are intended primarily to provide a richer understanding of participants’ questionnaire scores and changes over time, including participants’ experiences with the intervention, how they perceived changes in autonomy-connectedness, well-being, and ED recovery, and whether these experiences are reflected in quantitative assessments. In addition, the interviews will be used to explore the acceptability and feasibility of the intervention and to identify factors relevant to future implementation. As the qualitative component is exploratory and descriptive in nature, interview data will be reviewed and summarized to identify recurring experiences and implementation considerations rather than subjected to an in-depth qualitative analysis. For a study procedure flowchart, refer to Figure 1. The data will be analyzed using mixed models with an unstructured covariance matrix.

### Participants

Participants will be 22 to 30 patients (aged 18‐65 years) at the Center for Eating Disorders, GGZ Oost Brabant, Helmond, the Netherlands, in the last phase of multidisciplinary ED treatment, where treatment focuses on consolidating recovery, facilitating the transition to outpatient care or treatment completion, promoting self-management, and preventing relapse.

Prior to this last phase of treatment, participants will have completed 9 to 12 months of day care treatment for AN or BN, consisting of 2, 3, or 5 days of group therapy every week, including psychotherapy, sociotherapy, psychomotor therapy, drama therapy, and supported meals and snacks. All participants will have a BMI higher than 18.5 kg/m^2^.

All patients moving into the last phase of treatment are eligible for participation in this study. Inclusion and exclusion criteria for this study are therefore identical to the criteria for moving into the last phase of treatment. Eligibility for moving into the last phase of treatment is determined by the multidisciplinary treatment team. Patients enter this phase when ED symptoms are considered to be in remission at a behavioral level, allowing treatment to shift from symptom reduction toward maintaining recovery gains and preventing relapse. Main reasons for not progressing to the last phase include patients finding treatment closer to home, patients requiring specific care for posttraumatic stress disorder, autism, or personality disorders, or patients dropping out of treatment after failing to adhere to day care weight gain requirements.

### Procedures

All patients in day care ED treatment at GGZ Oost Brabant are informed about the study by the main practitioner and the principal investigator, both orally and in written form. Before deciding to participate, patients have opportunities to ask questions and have 2 weeks to consider participation. Those who agree to participate fill out an informed consent form on paper. Each set of 4 participants is randomly allocated without replacement to a baseline length of 5, 6, 7, or 8 weeks, using a predetermined random list of 150 numbers generated using statistical software by an independent person in the presence of the main investigator. To start participants on their baseline measurements on time, main practitioners are asked to estimate when patients are likely to move on to the last phase of treatment. When participants have started baseline measures but need more time in day care treatment, baseline measures are paused until they are close to moving on.

When participants have started baseline measures but choose not to continue into the last phase of treatment, their allocated baseline number is placed back on top of the list.

All questionnaires are answered digitally on any device. Weekly measures will take approximately 10 minutes; 3-monthly measures will take approximately 30 minutes. Questionnaire invitations as well as reminders are sent by email.

Posttreatment interviews will take place at the GGZ facility or online, conducted by the investigator and guided by a topic-list. Interviews are audio-recorded and later transcribed.

### Intervention

Autonomy-enhancing therapy’s goal is to enhance autonomy-connectedness at behavioral, attitudinal, and emotional levels by working on personal, autonomy-related goals.

The investigational treatment, autonomy-enhancing therapy, consists of 15 weekly 2-hour group sessions, following the existing autonomy-enhancing therapy protocol [21]. The autonomy-enhancing therapy protocol prescribes a mindfulness exercise, space to share progress toward personal autonomy goals, and the appointment of a chairperson for each session who, with therapist support, keeps time. Each week features different autonomy-related themes (eg, relationships, boundaries, body and sexuality, and emotions), including psychoeducation, exercises, homework, and discussion of autonomy-related difficulties. Autonomy-enhancing therapy groups typically consist of 8 participants (range 4‐10).

The autonomy-enhancing therapy protocol has been slightly adjusted to fit the existing structure of the last phase of treatment in the Center for Eating Disorders, GGZ Oost-Brabant, Helmond. In this phase of treatment, participants are part of a group that follows several treatment modules together. Relapse prevention is covered in standard treatment, so introductory, relapse prevention, and midpoint evaluation sessions are omitted, leaving 12 sessions. Additionally, new members continuously join the group in the last phase of treatment, so patients start with whatever theme is planned for that week, ensuring that all themes are covered, even if the starting point varies. In the last phase of treatment, patients participate for a minimum 3 to maximum 6 months, with continuation decided after an evaluation at 3 months. Continuation or discharge is determined by main practitioners, guided by input from the treatment team. Participation in the intervention stops when individuals are discharged. After finishing their ED treatment, many patients proceed to other forms of treatment (eg, trauma treatment, treatment focused on personality disorders, or general psychological support).

Because autonomy-enhancing therapy is embedded in treatment as usual (TAU) during the last phase of treatment, participants will receive 12 to 24 weeks of autonomy-enhancing therapy (Figure 1), ensuring that each patient covers every theme at least once and at most twice. As this study investigates the real-life application of autonomy-enhancing therapy in ED aftercare, all patients will also continue to receive TAU for the last phase of treatment, consisting of drama therapy, psychomotor therapy, and regular group therapy. TAU focuses on consolidating recovery gains, preventing relapse, and supporting the transition to everyday life.

Therapists providing autonomy-enhancing therapy are trained and certified in the autonomy-enhancing therapy protocol through RINO Amsterdam, a Dutch postgraduate training institute for mental health professionals. As part of maintaining certification, therapists participate in regular intervision meetings in which treatment delivery and adherence to the protocol are discussed. Formal assessment of treatment fidelity is not conducted within the present study. No major risks beyond regular treatment are expected. Any adverse psychological events, treatment deterioration, or participant withdrawal related to study participation will be discussed within the clinical treatment team and documented.

### Measures

Gender was, in a conventional way, assessed as biological sex as “woman” or “man.”

#### Primary Outcome Measures

Participants fill out a set of questionnaires regarding their weekly levels of autonomy-connectedness and ED symptoms.

To assess ED symptoms, the EDE-QS [36] is used. The EDE-QS is a brief, reliable, and valid measure of ED symptom severity. It consists of 12 items and was developed for routine, including session-by-session and outcome assessment, and it performs similarly to the Eating Disorder Examination Questionnaire (EDE-Q), which lends itself to the use of sessional outcome monitoring in treatment and research [37].

To assess weekly autonomy-connectedness levels, the Autonomy-Connectedness Scale (ACS-30) [38] was amended to allow for measuring short-term changes as the ACS-30-ST. The ACS-30-ST instruction asks about participants’ experiences during the past week (instead of “usually,” as in the original ACS-30). Existing questions have been rephrased where timing is concerned (eg, “I often long for…” has been changed to “In the past week I have longed for…”). The ACS-30-ST has been reviewed by a convenience sample to assess acceptability and feasibility, after which a few changes were made to questions that were hard to answer on a weekly basis (eg, “When making important decisions about my life, I leave other peoples’ opinions and wishes out of consideration” was changed to “When making decisions I leave other peoples’ opinions and wishes out of consideration.”) This process was repeated until the questionnaire was easy to use. Psychometric properties of the ACS-30-ST will be evaluated over the course of this study.

#### Secondary Outcome Measures

Every 3 months, participants fill out a longer set of questionnaires regarding their long-term autonomy-connectedness, ED pathology, depression, anxiety, self-esteem, and quality of life.

To assess autonomy-connectedness, the original ACS-30 is used [38]. This 30-item scale includes 3 subscales, self-awareness, sensitivity to others, and capacity for managing new situations. Items are rated on a 5-point Likert scale ranging from 1 (“disagree”) to 5 (“agree”). The reliability and validity are reported to be good [33].

To assess ED pathology, the EDE-Q is used [37]. This 22-item scale has been widely used to assess the frequency of disordered eating attitudes and behaviors, including restrictive eating, binge eating, and compensatory behaviors, occurring over the last 28 days. Items are scored on a 7-point scale ranging from 0 (no days or not at all) to 6 (every day or markedly). The scale includes 4 subscales: restraint (dietary restriction and attempts to limit food intake), eating concerns (preoccupation with eating and eating-related distress), shape concerns (concerns about body shape), and weight concerns (concerns about body weight). Internal consistency and 2-week test-retest reliability were excellent [37], and the global score showed strong discriminative validity in distinguishing individuals with an ED from those without [39].

To assess comorbid psychological symptoms, the depression and anxiety subscales of the Symptom Checklist-90 (SCL-90) are used [40]. The SCL-90 is a widely used measure of psychological distress with established reliability and validity in clinical and nonclinical populations [41].

To assess self-esteem, the Rosenberg Self-Esteem Scale (RSES) is used [42]. The RSES consists of 10 items and assesses global self-esteem. All items, containing positive and negative statements about the self, are rated on a 4-point Likert scale ranging from “strongly agree” to “strongly disagree.” The reliability and validity of the RSES are reported to be good [42].

To assess quality of life, the World Health Organization Quality of Life–Brief Version (WHOQOL-BREF) is used [43], which consists of 26 items, including 4 quality of life domains: physical health, psychological health, social relationships, and environment. Additionally, 2 items examine general quality of life. All items are rated on a 5-point Likert scale with 0 meaning “not at all,” “never,” “very poor,” or “very dissatisfied,” and 4 meaning “completely,” “always,” “very good,” or “very satisfied.” Psychometric properties have been studied in psychiatric outpatients and are reported to be good [44].

After treatment, an interview will be conducted to examine participants’ experiences with autonomy-enhancing therapy. The interview guide can be found in Multimedia Appendix 1.

### Analyses

Sample size and statistical power were approximated using G*Power (version 3.1.9.4). To detect a medium effect size (*f*^2^=0.15) in a regression with 7 predictors, 89 independent observations are needed for a power of 0.95 at α=.05. For the SCED analysis, with an intraclass correlation coefficient of 0.2, each participant’s 20 observations yield 4.17 independent observations [45], necessitating at least 22 participants. To ensure robustness, we aim for 30 participants (125 independent observations), achieving a power of 0.99. Considering a 20% dropout rate, we plan to recruit 36 participants. For the RM analysis, assuming 3 observations per participant, we obtain 30×2.14=64 independent observations, resulting in a power of 0.67 to detect a medium effect size (using α=.01).

Differences between individuals in their SCED data will be examined using visual analyses, presenting separate plots for all participants’ measures [46]. Visual analysis will evaluate changes in level, trend, variability, immediacy of effect, overlap between phases, and consistency across participants. Interview data will be used descriptively to better understand participants’ experiences of the intervention and their interpretation of change. The interview data will be reviewed and summarized to identify recurring experiences, perceived benefits and challenges, and considerations relevant to feasibility and future implementation.

The SCED data will be analyzed using a mixed model with 2 treatment periods, incorporating 2 dummy variables (treatment period 1 and treatment period 2, with baseline as the reference). A linear effect of time, centered within each period, represents the average change across periods. A random intercept and an autoregressive error structure will model the covariance structure. The primary hypothesis of the average decrease in ED symptoms is tested with 1 dummy for treatment period (1=treatment period 1 or 2, 0=baseline). This hypothesis will be tested for autonomy-connectedness.

The RM data for all outcome variables will be analyzed using mixed models. Measurement will be modeled with 3 dummy variables (after treatment period 1, after treatment period 2, and after 3 months, all with baseline as the reference category), and an unstructured covariance matrix will model the random effects. The analyses will focus on the effect of dummy “post,” testing the hypothesis that scores improve compared with baseline. Mediation by autonomy-connectedness will be tested in two steps: (1) adding autonomy-connectedness to the model and (2) conducting a dependent-samples *t* test (1-tailed) comparing autonomy-connectedness after treatment and at the 3-month follow-up with baseline. Mediation will be concluded if (1) autonomy-connectedness affects the outcome variable in the mixed model analysis and (2) autonomy-connectedness improves over time in the *t* test, indicating a joint-significance test of mediation [47].

All analyses will control for ED diagnosis (dummy AN, with BN as the reference category). We will explore whether the development over time of ED, autonomy-connectedness, and the other outcome variables differ between the two EDs.

Hypotheses on change will be tested 1-tailed, with a significance level of .05 for the multiple baseline data and .01 for the RM data to correct for multiple testing. Missing values on the outcome variable do not affect mixed model analyses [45], whereas participants with missing autonomy-connectedness values will be excluded from the mediation analyses.

## Results

Data collection started in March 2024. As of May 2026, 18 participants have completed the study, and 6 have been enrolled. An additional 7 participants have been recruited, whose participation will depend on their individual treatment paths. Data collection is projected to finish in February 2027; results are expected to be published in the second half of 2027.

## Discussion

This study addresses the need for better treatments for ED by testing the effectiveness of autonomy-enhancing therapy, a potentially useful addition to ED treatment. On the basis of previous research on autonomy-connectedness and autonomy-enhancing therapy in other clinical populations [12,23], we hypothesize that autonomy-enhancing therapy will contribute to reductions in ED symptoms and improvements in autonomy-connectedness, self-esteem, quality of life, depression, and anxiety. More specifically, we expect increases in self-awareness and the capacity for managing new situations, alongside decreases in sensitivity to others, to contribute to treatment success. If supported, the findings may provide further evidence for the role of autonomy-connectedness as an underlying vulnerability and maintenance factor in ED. Additionally, this study will provide information regarding the acceptability and feasibility of implementing autonomy-enhancing therapy within routine ED treatment, which will inform future effectiveness research and broader implementation.

This study builds on previous findings suggesting that autonomy-connectedness predicts recovery from ED treatment [12], and that disturbances in self-functioning and interpersonal functioning play an important role in ED psychopathology. Existing ED treatments primarily focus on symptom reduction, while difficulties related to identity, self-definition, autonomy, and interpersonal functioning may remain insufficiently addressed. Autonomy-enhancing therapy may therefore complement existing approaches by targeting these broader underlying processes.

A particular strength of this study is its practice-based design within a specialized ED treatment setting, enhancing ecological validity and clinical applicability. In addition, the SCED multiple baseline design enables insight into the treatment process, as opposed to the “black box” effect of a randomized controlled trial.

Several limitations should also be considered. First, participants will continue to receive TAU alongside autonomy-enhancing therapy, making it difficult to determine whether observed changes can be uniquely attributed to autonomy-enhancing therapy. However, because autonomy-enhancing therapy is intended as an adjunctive intervention integrated into routine clinical care, studying it within the context of ongoing treatment may also increase the clinical relevance of the findings. Second, the relatively small sample size may limit generalizability. Third, highest relapse risk is reported to occur approximately 4 to 6 months after treatment, whereas the current study includes a follow-up assessment at 3 months after treatment due to feasibility constraints. This study may therefore not fully capture relapse during the period of highest risk. Finally, recruitment and adherence may prove challenging due to the repeated weekly measures.

Future research should further examine the effectiveness of autonomy-enhancing therapy in larger and more diverse ED populations, investigate long-term outcomes, and examine effects on relapse. If the present study yields promising findings, future studies could include larger controlled trials to further evaluate efficacy.

The findings of this study will be disseminated through peer-reviewed publications and presentations at scientific conferences, as well as at practice-oriented conferences for clinicians working in ED treatment. In doing so, we aim to contribute to the development of more comprehensive and relapse-sensitive approaches to ED treatment.

## Supplementary material

10.2196/87349 Multimedia Appendix 1 Semistructured interview guide.

## Abbreviations

ACS-30: Autonomy-Connectedness Scale
AN: anorexia nervosa
BN: bulimia nervosa
ED: eating disorder
EDE-Q: Eating Disorder Examination Questionnaire
EDE-QS: Eating Disorder Examination Questionnaire–Short
RM: repeated measures
SCED: single-case experimental design
TAU: treatment as usual
WHOQOL-BREF: World Health Organization Quality of Life–Brief Version

## References

[1] Solmi M, Wade TD, Byrne S (2021). Comparative efficacy and acceptability of psychological interventions for the treatment of adult outpatients with anorexia nervosa: a systematic review and network meta-analysis. Lancet Psychiatry.

[2] Hilbert A, Hoek HW, Schmidt R (2017). Evidence-based clinical guidelines for eating disorders: international comparison. Curr Opin Psychiatry.

[3] Levels of evidence. Centre for Evidence-Based Medicine.

[4] Hay P, Chinn D, Forbes D (2014). Royal Australian and New Zealand College of Psychiatrists clinical practice guidelines for the treatment of eating disorders. Aust N Z J Psychiatry.

[5] Khalsa SS, Portnoff LC, McCurdy-McKinnon D, Feusner JD (2017). What happens after treatment? A systematic review of relapse, remission, and recovery in anorexia nervosa. J Eat Disord.

[6] Ágh T, Kovács G, Supina D (2016). A systematic review of the health-related quality of life and economic burdens of anorexia nervosa, bulimia nervosa, and binge eating disorder. Eat Weight Disord.

[7] Bardone-Cone AM, Thompson KA, Miller AJ (2020). The self and eating disorders. J Pers.

[8] Bruch H (2001). The Golden Cage: The Enigma of Anorexia Nervosa.

[9] Jacquemot AM, Park R (2020). The role of interoception in the pathogenesis and treatment of anorexia nervosa: a narrative review. Front Psychiatry.

[10] Vartanian LR (2009). When the body defines the self: self-concept clarity, internalization, and body image. J Soc Clin Psychol.

[11] Lampard AM, Tasca GA, Balfour L, Bissada H (2013). An evaluation of the transdiagnostic cognitive‐behavioural model of eating disorders. Eur Eat Disord Rev.

[12] Kuipers GS, Hollander SD, van der Ark LA, Bekker MH (2017). Recovery from eating disorder 1 year after start of treatment is related to better mentalization and strong reduction of sensitivity to others. Eat Weight Disord.

[13] Bekker MH, van Assen M (2017). Autonomy-connectedness mediates sex differences in symptoms of psychopathology. PLoS One.

[14] Bekker MH, Croon MA (2010). The roles of autonomy-connectedness and attachment styles in depression and anxiety. J Soc Pers Relat.

[15] Bekker MH (1993). The development of an autonomy scale based on recent insights into gender identity. Eur J Pers.

[16] Chodorow N (1989). Feminism and Psychoanalytic Theory.

[17] Rutten EA, Bachrach N, van Balkom AJ, Braeken J, Ouwens MA, Bekker MH (2016). Anxiety, depression and autonomy-connectedness: the mediating role of alexithymia and assertiveness. Psychol Psychother.

[18] (2013). Diagnostic and Statistical Manual of Mental Disorders.

[19] Frostad S, Bentz M (2022). Anorexia nervosa: outpatient treatment and medical management. World J Psychiatry.

[20] Gregertsen EC, Mandy W, Serpell L (2017). The egosyntonic nature of anorexia: an impediment to recovery in anorexia nervosa treatment. Front Psychol.

[21] Bekker M, van Helsdingen MA, Rutten L, Kouwenhoven B (2016). Behandelprotocol Voor Autonomieversterkende Interventie [Book in Dutch].

[22] Alford BA, Gerrity DM (1995). The specificity of sociotropy-autonomy personality dimensions to depression vs. anxiety. J Clin Psychol.

[23] Kunst LE, Maas J, van Balkom AJ, van Assen MA, Kouwenhoven B, Bekker MH (2022). Group autonomy enhancing treatment versus cognitive behavioral therapy for anxiety disorders: a cluster-randomized clinical trial. Depress Anxiety.

[24] Bekker MH, Belt U (2006). The role of autonomy-connectedness in depression and anxiety. Depress Anxiety.

[25] Bieling PJ, Beck AT, Brown GK (2000). The Sociotropy–Autonomy Scale: structure and implications. Cognit Ther Res.

[26] Burke A, Haslam N (2001). Relations between personality and depressive symptoms: a multimeasure study of dependency, autonomy, and related constructs. J Clin Psychol.

[27] Fairbrother N, Moretti M (1998). Sociotropy, autonomy, and self-discrepancy: status in depressed, remitted depressed, and control participants. Cognit Ther Res.

[28] Franche RL, Dobson K (1992). Self-criticism and interpersonal dependency as vulnerability factors to depression. Cogn Ther Res.

[29] Robins CJ, Luten AG (1991). Sociotropy and autonomy: differential patterns of clinical presentation in unipolar depression. J Abnorm Psychol.

[30] Bachrach N, Bekker MH, Croon MA (2013). Autonomy-connectedness and internalizing-externalizing personality psychopathology, among outpatients: autonomy-connectedness and internalizing-externalizing psychopathology. J Clin Psychol.

[31] Gorrell S, Hail L, Reilly EE (2023). Predictors of treatment outcome in eating disorders: a roadmap to inform future research efforts. Curr Psychiatry Rep.

[32] Levinson CA, Cusack C, Brown ML, Smith AR (2022). A network approach can improve eating disorder conceptualization and treatment. Nat Rev Psychol.

[33] Bekker MH, van Assen MA (2008). Autonomy-connectedness and gender. Sex Roles.

[34] Altemus M, Sarvaiya N, Neill Epperson C (2014). Sex differences in anxiety and depression clinical perspectives. Front Neuroendocrinol.

[35] Tomba E, Tecuta L, Crocetti E, Squarcio F, Tomei G (2019). Residual eating disorder symptoms and clinical features in remitted and recovered eating disorder patients: a systematic review with meta‐analysis. Int J Eat Disord.

[36] Gideon N, Hawkes N, Mond J, Saunders R, Tchanturia K, Serpell L (2016). Development and psychometric validation of the EDE-QS, a 12 item short form of the Eating Disorder Examination Questionnaire (EDE-Q). PLoS One.

[37] Luce KH, Crowther JH (1999). The reliability of the Eating Disorder Examination-Self-Report Questionnaire version (EDE-Q). Int J Eat Disord.

[38] Bekker MH, van Assen MA (2006). A short form of the Autonomy Scale: properties of the Autonomy-Connectedness Scale (ACS-30). J Pers Assess.

[39] Aardoom JJ, Dingemans AE, Slof Op’t Landt MC, Van Furth EF (2012). Norms and discriminative validity of the Eating Disorder Examination Questionnaire (EDE-Q). Eat Behav.

[40] Derogatis LR (1994). Symptom Checklist-90-R: Administration, Scoring & Procedure Manual for the Revised Version of the SCL-90.

[41] Arrindell WA, Ettema JH (2003). SCL-90: Handleiding bij een Multidimensionele Psychopathologie-Indicator [Book in Dutch].

[42] Sinclair SJ, Blais MA, Gansler DA, Sandberg E, Bistis K, LoCicero A (2010). Psychometric properties of the Rosenberg Self-Esteem Scale: overall and across demographic groups living within the United States. Eval Health Prof.

[43] (1998). The World Health Organization Quality of Life assessment (WHOQOL): development and general psychometric properties. Soc Sci Med.

[44] Trompenaars FJ, Masthoff ED, Van Heck GL, Hodiamont PP, de Vries J (2005). Content validity, construct validity, and reliability of the WHOQOL-BREF in a population of Dutch adult psychiatric outpatients. Qual Life Res.

[45] Snijders TA, Bosker RJ (1999). Multilevel Analysis: An Introduction to Basic and Advanced Multilevel Modeling.

[46] Morley S (2017). Single Case Methods in Clinical Psychology: A Practical Guide.

[47] MacKinnon DP, Lockwood CM, Hoffman JM, West SG, Sheets V (2002). A comparison of methods to test mediation and other intervening variable effects. Psychol Methods.

