# Supplementary material for: The Effects of Autonomy-Enhancing Therapy for Eating Disorders: Protocol for a Single-Case Experimental Multiple Baseline Design
Source: JMIR Res Protoc. 2026 Sep 22;15:e87349. doi: 10.2196/87349 (PMC13597025; doi:10.2196/87349)
Supplement: Supplementary file 1 [file resprot-v15-e87349-s001.docx]

**Appendix A: Interview guide**

**Semi-Structured Interview on AET in ED**

**Introduction**

Thank you for participating in this interview.

My name is -.

**Purpose of the Interview: Semi-Structured Interview on AET at ED**

We’d like to know what you thought about the autonomy module. The purpose of this interview is to gain more insight into your personal experiences so that we can better understand the results of the questionnaires. It’s important that you answer the questions as honestly and completely as possible. I’ll be recording the conversation; the recording and all your answers will be treated confidentially. You can ask questions or interrupt the conversation at any time.

**Introduction to autonomy-attachment:**

Autonomy-attachment is about the balance between being independent and having healthy relationships. People want to be both independent and connected to others. These two things do not have to be in conflict; you can be independent while still finding support and warmth in your relationships. To achieve this, you need to have a clear understanding of what you want, feel, and need. You must be able to communicate this to others while also being attuned to what the other person wants. But also not overly so, to the point that you simply go along with whatever the other person wants or let yourself be overly influenced.

**Acceptability**

1. What did you think of the autonomy module?

● What aspects did you find useful, and what did you find less useful?
● Are there any topics that really belonged here but weren’t included yet?
● Are there any other things you would like to change?

  2. What did you think of the timing of the autonomy module within the treatment program?

  3. How did you find working with the autonomy therapists? How was your interaction with them?

  4. Compared to the other therapy modules, what does this module add?

  **Change of symtpoms**

  [Instruction for interviewer: Have pre- and post-scores for each construct ready; discuss the differences.]

  1. How would you describe your relationships with significant others, and how have they changed?
● Has completing the autonomy module had any effect on this?
● If yes, which aspect of the autonomy treatment was responsible?

  2. How would you describe your eating behavior? (EDEQ)
● Did participating in the autonomy module have any effect on this?
● If yes, which aspect of the autonomy treatment was it?

  3. How would you describe your sensitivity toward others? (SO)
● Has taking the autonomy module brought about any changes in this regard?
● If yes, which aspect of the autonomy treatment was that?

  4. How would you describe your self-awareness? (SA)
● Has anything changed in that regard since you took the autonomy module?
● If yes, which aspect of the autonomy treatment was that?

  5. Can you tell me something about how you deal with new situations? (CMNS)
● Has anything changed in that regard since you took the autonomy module?
● If yes, which aspect of the autonomy treatment was that?

  6. Can you tell me something about how you view yourself? (RSES)
● Has anything changed in that regard since you started the autonomy module?
● If yes, which aspect of the autonomy treatment was that?

  7. Can you tell me a little about your quality of life? (WHOQOL)
● Has anything changed in that regard since you started the autonomy module?
● If yes, which aspect of the autonomy treatment was it?

  8. How have any other mental health issues you have changed since you joined the group? [Particularly ANXIETY & DEPRESSION]
● If yes, which aspect of the autonomy treatment was that?

  9. You’ve already mentioned a lot of things—are there any other changes you noticed during the autonomy module, and what do you think are the reasons for them?

  **Personal Goals**

  1. Can you tell us about your goals in the autonomy module? To what extent do you feel you’ve achieved those goals, and how exactly?

  2. How did you see the connection between autonomy and your eating disorder? Did autonomy play a role in your ED? And how do you view that connection/role now?

  3. What were your thoughts on autonomy and connectedness before you joined the group? And after the module?

  **Closing Questions**

  Are there any other issues you’d like to discuss that haven’t been addressed yet?
